# Editorial: State of the science of pharmacogenomics implementation in healthcare systems and communities

**DOI:** 10.3389/fphar.2024.1463384

**Published:** 2024-07-24

**Authors:** Sean P. David, Beth Devine, Latha Palaniappan, Elisa J. Houwink

**Affiliations:** ^1^ Department of Family Medicine, Pritzker School of Medicine, University of Chicago, Chicago, IL, United States; ^2^ Endeavor Health, Center for Personalized Medicine and Department of Family Medicine, Evanston, IL, United States; ^3^ School of Pharmacy, Biomedical Informatics and Medical Education, University of Washington, Seattle, WA, United States; ^4^ Department of Medicine, Stanford University School of Medicine, Cardiovascular Medicine, Stanford, CA, United States; ^5^ Department of Family Medicine, Mayo Clinic College of Medicine and Science, Rochester, MN, United States

**Keywords:** pharmacogenomics, pharmacogenetics, implementation, community, global, education

**FIGURE 1:**
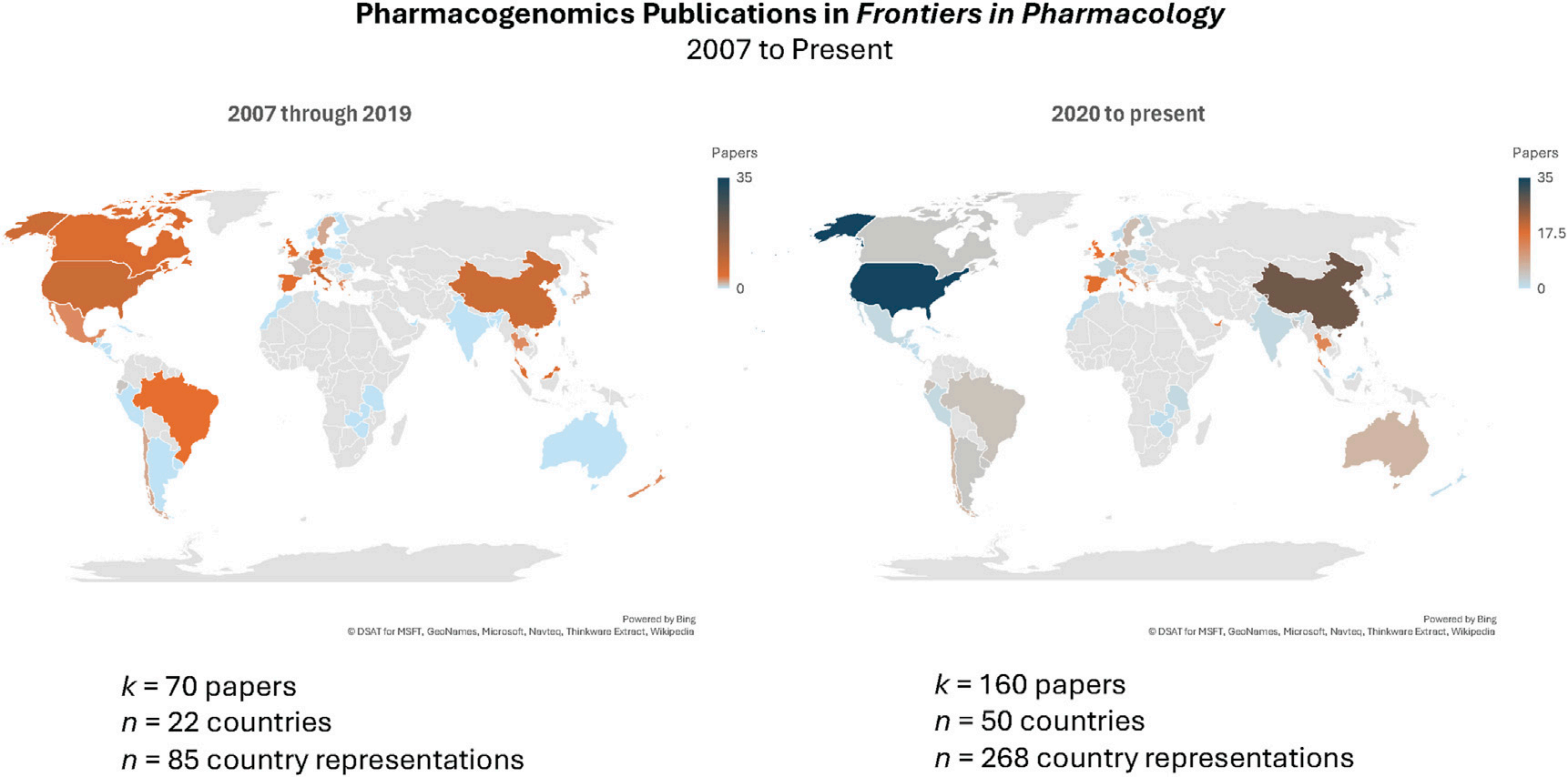
Publication search of *Frontiers in Pharmacology* papers “Pharmacogenetics or Pharmacogenomics” conducted on 3 May 2024 (https://www.frontiersin.org/articles?publication-date=01%2F01%2F2007-04%2F05%2F2024&query=pharmacogenetics%20or%20pharmacogenomics&sort=1&journal=176) yielded 257 papers; 27 papers excluded after screening because of non-pharmacogenomics-related scientific content. Heat map represents number of papers published by authors from that country.

This Research Topic on the “*State of the science of pharmacogenomics implementation in healthcare systems and communities*” offers a horizon scan of early adopter programs that have expanded beyond the bricks and mortar of clinics and hospitals to expand the reach of pharmacogenomics (PGx) across multiple countries and geographic regions, including rural health networks. These diverse papers delve into frontiers in medical and pharmacy education and patient advocacy and explore in real-world systems the impact of testing on clinical outcomes. As illustrated in Figure 1, *Frontiers in Pharmacology* publications on PGx have more than doubled, with a more than 200% increase in nations represented since 2020 compared to all prior years in the history of the Journal. Here, we feature the implementation journeys of five diverse programs across multiple countries and continents. We hope that the increasing diversity of global implementation efforts will optimize the utility of pharmacogenomic applications to all populations.

Wu et al. describe a large-scale program that offered *DPYD* variant testing to all cancer patients treated with fluoropyrimidine medications first in the British Columbia (BC) Cancer Center in Vancouver and then across the entire Canadian province. Fluoropyrimidines are widely used to treat a range of solid tumor types, including breast, colorectal, gastric, head and neck, and pancreatic cancers. It has been widely documented and established that patients with *DYPD* genetic variants that portend reduced activity of the 5-FU inactivating enzyme dihydropyrimidine dehydrogenase (DPD) are at markedly higher risk for fluoropyrimidine-related toxicity. Frequently occurring fluoropyrimidine-related adverse events include diarrhea, hand-foot syndrome, mucositis, myelosuppression, nausea, and vomiting. However, DPYD-guided dose reductions allowed many patients to be treated safely with fluoropyrimidines and avoid related toxicity. The implementation of this project was deemed feasible and scalable over 9 months. It informeded the debate about whether preemptive DYPD variant testing to guide fluoropyrimidine prescribing should be the standard of care internationally.

Van Heukelom et al. described the successful implementation of a multi-professional PGx clinic that served in-person and virtual patients across a large rural region of four upper Midwest states in the United States (US). The pharmacogenomic clinic was implemented to fill a gap in provider coverage and enable expert teams of pharmacists and advanced practice providers (APP) to provide pre- and post-test genetic counseling and PGx medication recommendations before and after testing. The article discusses the evolution of Sanford Health’s PGx programs, which started with implementing CYP2C19-guided antiplatelet therapy and progressed to developing the “Sanford Chip.” The program includes in-person and virtual services, provider and patient education, and a referral process mainly for primary care and subspecialty referrals. The authors claim their success lies in using technology to serve a large geographical area with a small team. The care model is transportable to other health systems and geographical regions.

In contrast to the Canadian province-wide and US-based extensive rural network PGx implementation programs described above, Stewart et al. describe a retrospective descriptive analysis of 10 years of activities using annual data from the Clinical Pharmacogenetics Unit within the La Paz University Hospital, a 1,300-bed tertiary hospital in Madrid, Spain. The authors’ primary objective was to describe the implementation experience of the Clinical PGx Unit over 1 year. They also conducted a head-to-head comparison of activity results between two periods from 2014 through 2016 (period 1) and 2021 to 2022 (period 2) to capture temporal trends in testing activity. The analyses indicate substantial utilization of pharmacogenomic testing spanning a range of specialists (17 departments) ordering the tests and a 35% growth rate in PGx testing over time. The majority (58%) of test orders came from oncologists, and the dominant preemptive genotyping tests ordered were for the chemotherapy-related gene-drug pairs–*DYPD*/fluoropyrimidines (34%) and *UGT1A1*/irinotecan (29%). The decade-long preemptive genotyping experience of this large, metropolitan tertiary hospital in Madrid presented data that the authors claim “strongly endorse the integration of PGx testing into everyday clinical practice” across real-world health systems.

In a study to adopt the use of PGx in future clinical practices of students at the University of Sharjah in Abu Dhabi, United Arab Emirates (UAE), Al-Suhail et al. from the UAE, Greece, and the Netherlands developed a questionnaire based on the Theory of Planned Behavior to evaluate the impact of different factors on students’ intentions. The questionnaire was distributed to 467 students from all academic years, including medical, dental, nursing, and pharmacy students, over 3 months in late 2022. Sixty percent of students who responded demonstrated a high level of knowledge. They agreed that PGx testing can improve patient safety—more than two-thirds of students intended to promote PGx adoption in their future clinical practice. However, only half of the students expressed confidence in their abilities to counsel patients on test results. Students’ most significant perceived barriers to implementation were test reimbursement, privacy concerns, and lack of sufficient trained personnel to support PGx clinical services. Overall, students rated a high degree of positive intentions to adopt PGx in the future.

Ferwerda et al. conducted focus groups to identify the perceived role of PGx in primary care practice, knowledge gaps, and necessary skills. Six focus groups were held, and four themes emerged: the need for greater competency in PGx clinical care, clarity about the roles of different health professionals in providing PGx services, workflow concerns with electronic medical records, and concerns about cost, equity, and psychological effects of testing. The authors suggest that addressing these themes and developing educational models can better prepare primary care physicians and other healthcare professionals to adopt PGx testing.

Together, this Research Topic of articles highlight the feasibility of cross-border partnerships and provide examples of successful collaborations that are crucial for addressing PGx implementation challenges and ethical implications in global healthcare systems. Working together, we can create a responsible ecosystem that benefits individuals and society. By outlining a path for secure data flow, efficient variant testing with clinical decision support, and measuring patient-centered medical outcomes, we can use PGx data to its full potential.

